# Changes of Plasma Fatty Acids in Four Lipid Classes to Understand Energy Metabolism at Different Levels of Non-Esterified Fatty Acid (NEFA) in Dairy Cows

**DOI:** 10.3390/ani10081410

**Published:** 2020-08-13

**Authors:** Rossella Tessari, Michele Berlanda, Massimo Morgante, Tamara Badon, Matteo Gianesella, Elisa Mazzotta, Barbara Contiero, Enrico Fiore

**Affiliations:** Department of Animal Medicine, Production and Health, University of Padova, Viale dell’Università 16, 35020 Legnaro, Italy; rossella.tessari@unipd.it (R.T.); michele.berlanda@unipd.it (M.B.); massimo.morgante@unipd.it (M.M.); tamara.badon@unipd.it (T.B.); matteo.gianesella@unipd.it (M.G.); elisa.mazzotta@unipd.it (E.M.); barbara.contiero@unipd.it (B.C.)

**Keywords:** transition period, non-esterified fatty acid, β-hydroxybutyrate, fatty acids, lipid class, hyperketonemia

## Abstract

**Simple Summary:**

Dairy cows in the transition period require energy for fetal growth and milk production. In this phase, energy requirement exceeds the amount available for the animal, developing a negative energy balance. Numerous metabolic processes are involved to improve the energy requirement, in particular the mobilization of adipose tissue occurs. Dairy cows with an inadequate adaptive response to the negative energy balance can develop metabolic diseases such as subclinical ketosis. The purpose of the present study was to identify new biomarkers among the plasma fatty acids (FAs) through the use of thin layer chromatography and gas chromatographic techniques (TLC-GC). Early detection of excessive lipomobilization could improve animal health and reduce economic losses on farms. The current study determined the FA concentrations of four plasma lipid classes in plasma, in two groups of cows with different degrees of lipid mobilization in order to obtain biomarker for an early diagnosis of metabolic diseases.

**Abstract:**

The transition period is a central moment in dairy cows breeding because metabolic disorders may occur in relation to a dramatic increase in energy demand. This research aimed to identify new biomarkers for the diagnosis of hyperketonemia in bovine in early lactation phase with different value of plasmatic non-esterified fatty acid (NEFA). The profile of plasma fatty acids (FAs) divided into four lipid classes was evaluated using thin layer chromatography and gas chromatographic techniques (TLC-GC). A group of 60 multiparous Holstein–Friesian dairy cows were recruited in the present study. Blood samples were collected from the coccygeal vein and NEFA and the β-hydroxybutyrate (BHB) were evaluated. All animals were divided in 2 groups based on NEFA, NEFA0 group had as mean value 0.24 ± 0.12 mEq/L and NEFA1 group had as mean value 0.87 ± 0.23 mEq/L. Plasma FA concentrations were analyzed separately in free fatty acids, cholesterol esters, phospholipids and triglycerides. Six FAs demonstrated a predictive value in the hyperketonemic dairy cows. In the free fatty acid class, the predictive FAs were C14:0 (AUC = 0.77), C18:1 ω 9 (AUC = 0.72), C18:1 ω 7 (AUC = 0.70) and C18:3 ω 3 (AUC = 0.68). In the phospholipids class the predictive parameters were C12:0 (AUC = 0.78) and C8:0 (AUC = 0.73). In cholesterol, esters and triglycerides lipidic classes no FA had a predictive function.

## 1. Introduction

The transition period is the interval that starts three weeks before calving and ends at three weeks after parturition [1]. The term transition underlines the strong metabolic change, physiological and nutritional, where energy requirements (needs for fetal growth and milk production) exceed energy intake [2,3,4]. This disequilibrium is expressed in a negative energy balance (NEB) further aggravated by the reduction of dry matter intake (DMI) [5].

The growing energy demand in cows with high milk production was satisfied through lipid mobilization. This inevitably causes a shift from anabolic to catabolic metabolism [6]. Triglycerides (TAG) are the main lipid molecules stored as an energy reserve in adipose tissue [7]. In NEB, TAG undergo a biochemical hydrolysis pathway mediated by hormone-sensitive enzymes called lipases (LPS). LPS activate the catabolic process that occurs in the release of high energy substrates into the bloodstream. TAG molecule releases one glycerol and 3 non-esterified fatty acid (NEFA) molecules which are the major source of energy for the tissues during periods of NEB [4,8].

The profile of plasma NEFA may reflect the composition of adipose tissue in terms of FAs. Indeed, the main FAs of the subcutaneous adipose tissue in periparturient cows are Stearic acid (C18: 0), Palmitic acid (C16: 0) and Oleic acid (C18: 1 ω 9) [9]. NEFAs in the bloodstream are associated with various plasma lipid classes and included NEFA, phospholipids (PL) and neutral lipids (NL). This last group is in turn divisible into 4 groups: cholesterol esters (CE), monoglycerides (MG), diglycerides (DG) and triglycerides (TG) [10].

The acidic profile of every plasma lipid fraction is different. Linoleic acid (C18:2 ω 6), C18:0, C16:0 and C18:1 ω 9 are the FAs with the greatest content in PL class. In CE fraction, C18:2 ω 6 and Linolenic acid (C18:3 ω 3) are the predominant FAs. Stearic, Palmitic and Oleic acids are the main constituents of both lipid class NEFA and TG. Stearic acid has a predominant role for both fractions [11].

In the transition period, the enhanced lipomobilization effects the concentrations of total plasma lipids and causes an important shift in FA composition of different fractions [10]. Moreover, in the transition period changes in the lipid profile composition are not well studied. 

NEFAs are biomarkers of excessive lipid mobilization in high milk production cows and indicate an increased risk of developing pathologies such as ketosis, abomasal dislocation, metritis and mastitis [12]. NEFA concentration above the threshold of 0.29 mEq/L in the last gestation phase is considered critical, whereas in the postpartum the cut-off of an increased risk of developing metabolic pathologies is greater than 0.57 mEq/L [13].

The 15–20% of the total NEFA in the blood is taken from the liver [8,14]. Into hepatocytes, NEFA are used for energy production through the Krebs cycle, can be re-esterified in triacylglycerols (TAG) and exported as very low-density lipoproteins (VLDL). Otherwise they can be converted into ketone bodies such as acetoacetate, β-hydroxybutyrate (BHB) and acetone [8,14,15]. Elevated plasma NEFA concentration could induce clinical or subclinical ketosis when enhanced gluconeogenetic process removes the oxalacetate to the Krebs cycle increasing the formation of ketone bodies [16,17]. Clinical ketosis in dairy cows usually develops between the second and the seventh week of lactation with a peak of occurrence in the fourth week [18,19].

The excessive rise of NEFA and BHB indicates a poor adaptive response to NEB and has a direct negative effect on the animal health. NEFA and BHB are therefore used as markers of metabolic diseases in the transition period [20].

Detection of new biomarkers for the diagnosis of subclinical and clinical ketosis using Thin Layer chromatography and gas chromatography techniques (TLC-GC) was the aim of our research study. TLC-GC allowed the purification of metabolites (FA) from the biological matrix (blood samples).

Metabolomics, an emerging field of chemical studies, uses many analytical techniques, including high performance liquid chromatography (HPLC), nuclear magnetic resonance spectroscopy (NMR) and gas chromatography-mass spectrometry (GC-MS), in order to determine the metabolites from biological samples and to identify biomarkers of disease [21]. TLC-GC could therefore be a new technique for the isolation of biomarkers as fatty acids present in the lipid fraction of plasma.

Therefore, the present study aimed to establish the influence of lipomobilization on acidic profiles and to evaluate the association between serum concentration of NEFA and the changes in the fatty acid profiles in a group of Holstein–Friesian dairy cows in early lactation using TLC-GC.

## 2. Materials and Methods

### 2.1. Animals and Blood Samples

Sixty Holstein–Frisian dairy cows were enrolled in early post-partum (26.5 ± 1.5 days in milk). The total mixed ration (TMR) and the chemical composition of lactation diet was previously reported in the study of Fiore et al. [22]. Blood samples, analyzed for this research, were the same collected in the study of Fiore et al., taken from the high producing dairy farm located in Padua, Italy (45°36′ N. 11°40′ E. 23 m above sea-level) [18,22,23]. 

Blood samples were collected from the coccygeal vein using a vacutainer system. The BHB was measured using the Nova Biomedical Express digital reader (Nova Biomedical, Runcorn, UK) with specific BHB test strips (Stat Strip Ket, Nova Biomedical, Runcorn, United Kingdom), before taking the blood sample.

The purpose of the measurement of ketone bodies in the farm was to select dairy cows with a concentration of BHB greater than 1.0 mmol/L. The quantity of ketone bodies above this numerical value is often present in asymptomatic animals, but it is an excellent indicator of the risk of developing metabolic disorder in the postpartum [13].

The blood samples were collected for each bovine enrolled. Two samples were collected in vacuum tubes containing EDTA (5 mL; Terumo Venoject, Leuven, Belgium) and one in Venosafe tubes containing Clot Activator (9 mL; Terumo Venosafe, Leuvel, Belgium). 

### 2.2. Blood Analysis

Serum biochemistry was performed by an automatic analyzer (BT3500 Biotecnica Instrument SPA, Rome, Italy) and serum NEFA concentration was measured with the NEFA RX Monza test colorimetric method (Randox, Crumlin, UK). Animals were divided in two groups on the basis of the levels of NEFA. The first group (NEFA0) had 41 animals with NEFA concentration lower than 0.56 mEq/L (healthy animals). The second group (NEFA1) had 19 animals with a NEFA quantity higher than 0.56 mEq/L (pathological/sick animals). The NEFA value used as cut-off was chosen based on the study of Ospina et al. This value indicates excessive lipomobilization and could be considered as a guideline for monitoring dairy cows at risk of postpartum diseases [13]. 

For each animal, 35 FA were obtained per four lipid classes: free fatty acids (FFA), phospholipids (PL), triglycerides (TG) and cholesterol esters (CE). The extraction of lipids from plasma, the separation of lipid classes by TLC, the methylation of the carbon chain of fatty acids and GC were performed as in accordance with the study of Fiore et al. [22].

### 2.3. Statistical Analysis

The statistical processing of the data was carried out using the SAS system software (version 9.4; SAS Institute Inc., Cary, NC, USA). General Linear Model (GLM) analysis aimed to highlight the different composition of fatty acids in the four lipid classes compared to the two different groups (NEFA0–NEFA1). The Boruta test (R2 software, Santa Monica, CA, USA) was performed to identify fatty acids that could have a greater importance inside the decision algorithm and in discriminating dairy cows in hyperketonemia. Subsequently, the Receiver Operating Characteristic (ROC) analysis was performed using MedCalc Software (Ostend, Belgium) to establish the cut-off on the plasma fatty acids considered predictive/diagnostic. Statistical analysis was further explained in the study by Fiore et al. [22].

## 3. Results

Table 1 summarizes the data of NEFA, BHB, day in milk (DIM), body condition score (BCS), parity and yield milk produced (kg/day) for the 60 dairy cows, divided on the basis of two classes of NEFA (NEFA0 vs. NEFA1). The mean values of NEFA were respectively 0.24 ± 0.12 mEq/L for NEFA 0 group and 0.87 ± 0.23 mEq/L for NEFA 1 group.

TLC-GC technique allowed to assess the composition of FA in terms of the four lipid classes of FFA, CE, PL and TG. The mean value of the 35 plasma FAs (±SD) relating to these four classes of lipids was compared between the two groups with different blood concentrations of non-esterified fatty acid (NEFA0–NEFA1).

The values of the different plasma FAs related to the lipid class of FFA, CE, PL and TG comparing the values in the two NEFA groups are presented in Table 2. In the lipid class of FFA, 12 fatty acids were significant (*p* ≤ 0.05) (Table 2). In the lipid class of CE only C20:3 ω 9 (*p* = 0.05) was a significant fatty acid. In the lipid class of PL four FA resulted significantly different in the two class of NEFA.

Results of the evaluation of the plasmatic fatty acids (FAs) of the TG class are presented in the Table 2 and two FA resulted significantly different in the two groups of NEFA.

### Predictive Fatty Acid and Cut-Off Related to Animals in Hyperketonemia

**Figure 1:**
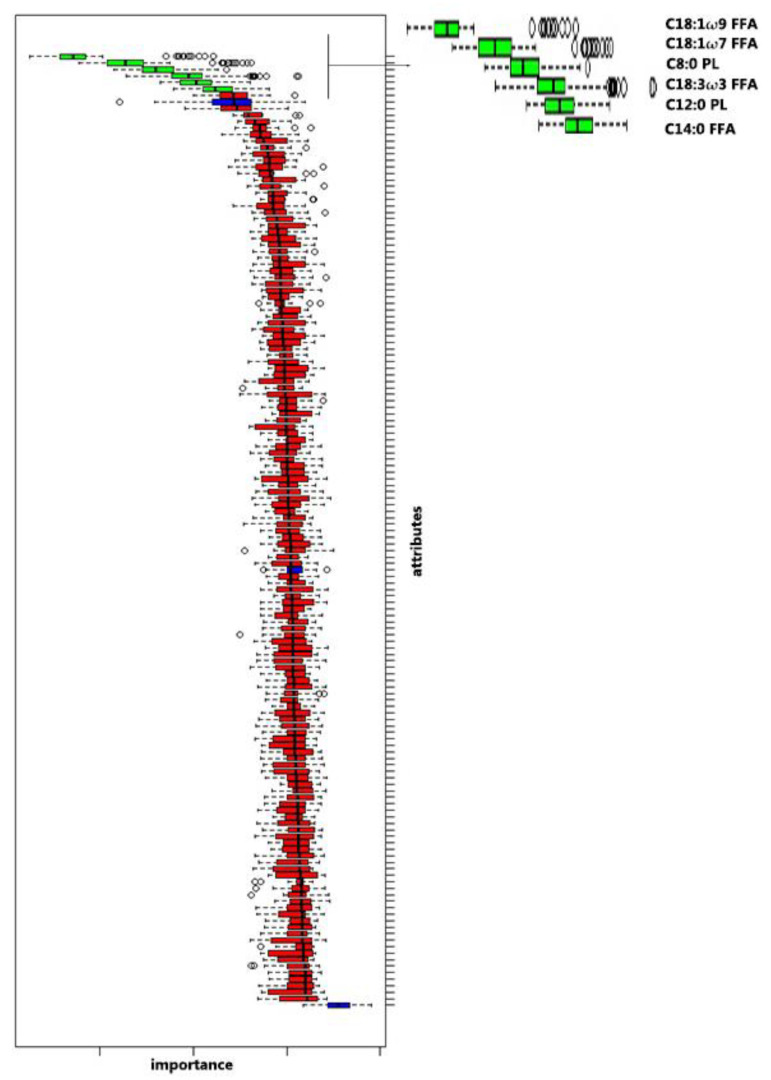
Box plot of Boruta decisional algorithm and detail of the green box plot with nomenclature of the main predictive fatty acids (FAs). Green box plot represented the fatty acid with the greatest predictive function. Red box plot represented the FAs with null predictive function.

The Boruta decisional algorithm identified FA with a predictive value. In Figure 1 are shown the six significant FAs.

Among the six predictive FAs, four (C14:0, C18:1 ω 9, C18:1 ω 7 and C18:3 ω 3) belong to the FFA class while the remaining two (C12:0 and C8:0) are included in the PL class. No plasmatic fatty acid belonging to the lipid class of CE and TG have been found as potential predictive parameter of lipid mobilization and HK. In the Table 3 are presented data relative to the six predictive FAs, listed in descending order of predictive value. 

**Figure 2:**
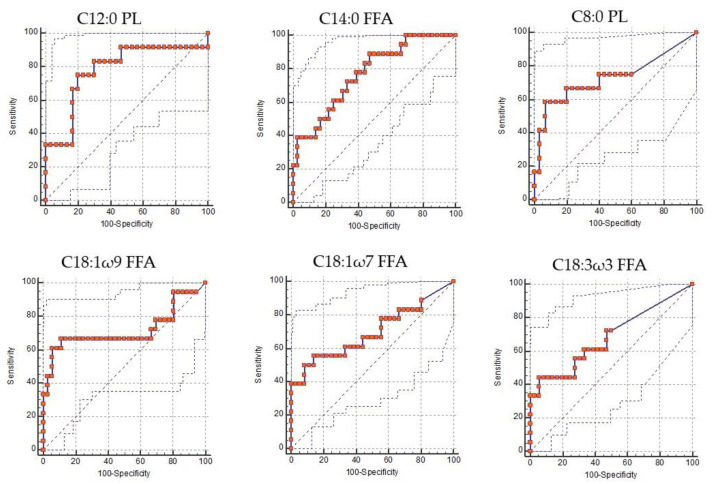
Receiver Operating Characteristic (ROC) curve of six predictive fatty acids with an area under the curve (AUC) higher than 0.70.

The Receiver Operating Characteristic curve analysis was performed on the six predictive FA (Figure 2) to establish the cut-off on the development of HK. The ROC curves were derived from the analysis of plasma data of all experimental animals (NEFA0–NEFA1) distinguishing sick animals (NEFA1) with the aim of identifying the limit beyond the animal was considered sick/hyperketonemic.

In order to identify plasma FA with moderate predictive function a threshold of AUC was set at 0.70.

Table 4 presents the data of the five FA selected on the basis of the 0.70 threshold. C18:3 ω 3 showed an AUC lower than 0.70 and was therefore eliminated.

## 4. Discussion

The data of this research belong to the “BovineOmics Project” (supported by University of Padua, Padova, Italy), based on the analysis through TLC-GC of the profile of plasma lipids in bovine during the transition period. In the study of Fiore et al., 2020, the data were previously analyzed evaluating blood BHB value whereas, in this study, the plasma concentrations of fatty acids were processed focused on the NEFA blood [22,23]. According to the literature, both NEFA and BHB are recognized as indicators of the risk of developing metabolic disorder in lactating period of dairy cows [12,24].

The threshold, that we established for the creation of the two groups, reflects the study of Ospina et al., where the NEFA cut-off value discriminating healthy animals from animals in a state of excessive lipomobilization in postpartum was equal to 0.57 mEq/L [13]. We used the same numerical value as threshold in order to discriminate healthy and sick animals.

Plasma concentrations of FA in NEFA0 and NEFA1 groups were not significantly correlated with DIM, BCS, parity and milk yield (Table 1).

The dairy cows with loss of a BCS points are more predisposed to the development of metabolic disease [25]. Moreover, plasma fatty acid concentrations increase more in postpartum dairy cows with a high BCS at the time of calving [26]. BCS drops abruptly the first 42 DIM retaining relatively constant thereafter in animals with normal concentration of BHB. In ketotic bovine, the BCS continues its negative decrease until it reaches the minimum score at 90 DIM [27]. Therefore, in our design, the absence of repeated measures of BCS could be a limitation of our work.

The enhancement of lipolysis and a decrease in lipogenesis resulted in a release of NEFA as consequence of the NEB [28]. The liver has a capacity to remove these molecules from the plasma and to employ them for energy production, for the formation of ketone bodies as BHB or to convert them into triacylglycerols (TAG) [15]. Blood BHB, an indicator of NEFA oxidation, is therefore positively correlated to the parameter used for the subdivision of the animals into the two groups NEFA 0 and NEFA 1 [13]. Currently, NEFA and BHB are biomarkers of the excessive lipid mobilization and of the diagnosis of HK in bovine peripartum [29,30,31].

In our research the BHB value is statistically significant (*p* = 0.001) with the variation of the blood NEFA as reported in the available literature. Indeed, postpartum NEFA concentrations greater than 0.4 mEq/L indicate an increased risk of developing uterine diseases and metabolic disorders such as ketosis [32]. 

The NEFA0 group, including healthy animals, had 0.24 ± 0.12 mEq/L as the mean value of the blood concentration of NEFA and the concentrations of BHB were equal to 0.65 ± 0.28 mmol/L. The NEFA1 group presented mean value of NEFA above the threshold limit of 0.57 mEq/L (0.87 ± 0.23 mEq/L) and for this reason we considered the animals enrolled in this group as sick animals. Furthermore, the mean value of BHB in NEFA1 group was 1.21 ± 0.28 mmol/L, an indicator of HK. In available literature, the diagnosis of subclinical ketosis is based on the BHB concentration between 1.0 mmol/L and 1.4 mmol/L [33].

We studied the concentrations of FA belonging to four plasma lipid classes as a function of two different concentrations of NEFA and therefore also two different BHB values (NEFA0 and NEFA1 group). The plasma fatty acids of the four fractions of FFA, CE, PL and TG changed in accordance with lipomobilization (Table 2). In previous studies is reported that ketotic bovine have a change in FA profile of the plasma lipid classes compared to healthy dairy cows [26,34,35].

### 4.1. Effect of Blood NEFA on Lipid Class of FFA

The function of adipose tissue is to store fat as an energy substrate in the form of TAG, which is used when required energy exceeds energy intake. The energy demand is guaranteed through the catabolic biochemical pathways such as proteolysis and lipolysis [14]. Yamdagni and Schultz showed that in HK dairy cows, the FFA concentrations were 10-fold elevated compared to the healthy subjects [34]. In our study, the total fatty acid concentration of the FFA class reflected what was found in literature, in fact, it was evident that the concentration was higher in the NEFA1 group (13.369 ± 10.591 mg/dL) compared to the NEFA0 group (5.960 ± 2.852 mg/dL) (Table 2) [34,36].

The FA, which most increase the plasma composition, are the same ones that are incorporated in adipose tissue, as evidence of the mobilization of lipid reserves in NEB [9,10,37].

The main FAs deposited in the adipocytes are C16:0, C16:1, C18:0 and C18:1 [26]. Indeed, during lipolysis, induced by NEB, there is a particular increase in the bloodstream of the C16:0, C18:0 and C18: 1 and Linoleic acids (C18: 2) [26,38]. As previously reported, the plasma acidic profile reflects the fatty acid composition of the fat reserves [9]. Dairy cows in early lactation phase, where the NEB is maximum, have higher concentrations and mobilization of FA than cows in medium or late lactation [39]. In our data the FA belonging to FFA lipid class, having *p* ≤ 0.05, shown the higher plasmatic concentration in hyperketonemic cows with more fat mobilization compared to healthy bovine (Table 2). 

Palmitic acid (*p* = 0.0011) in NEFA0 group had mean value of 1.365 ± 0.257 mg/dL, whereas in NEFA1 group the concentration had mean value of 2.908 ± 0.364 mg/dL. Stearic (*p* = 0.0019), Oleic (*p* = 0.0007), Linoleic (*p* = 0.0457) acids, as reported in the mentioned study, increased their concentration in hyperketonemic bovine (in NEFA1, respectively 2.315 ± 0.285 mg/dL, 3.241 ± 0.634 mg/dL and 0.475 ± 0.069) compared to healthy cow (NEFA0, respectively 1.176 ± 0.201 mg/dL, 0.456 ± 0.448 mg/dL and 0.302 ± 0.049 mg/dL). In the study of Contreras at al., C16:0 and C18:0 are the main FAs in the lipid class of FFA that increase at the moment of maximum lipid mobilization after calving [10]. C16:0 and C18:0 may be important regulators of metabolism and gene transcription in ruminants [37]. White et al. suggested that FFA plasmatic, in transition cows, contribute to increased gluconeogenesis and to maintain oxaloacetate for the tricarboxylic acid cycle [40]. 

In the class of FFA, twelve significant FA shown an increasing trend according to the increase of lipomobilization and therefore to the NEFA and BHB concentration (Table 2). Eight out of twelve FAs had unsaturated hydrocarbon chains. The plasma increase of unsaturated fatty acids (UFAs), belonging to the class of FFA, is attributed to the activity of hormone sensitive lipases (LPL) [41]. This enzyme has greater hydrolytic activity in triglycerides composed of unsaturated FA than in triglycerides consisting of saturated hydrocarbon chains [42]. The different activity of the LPL derives from the higher partition coefficient of the PUFA compared to the SFA.

A greater partition coefficient is expressed in a better lipase activity at the interface between lipid and aqueous solution and therefore, a greater release of UFAs [42]. Furthermore, in available literature it is known that UFAs increase the concentrations in the state of HK [35]. 

### 4.2. Effect of Blood NEFA on Lipid Class of CE

Dairy cows with high concentrations of ketone bodies in the blood present a CE value reduced by 34% compared to healthy animals [34]. The plasma CE concentration depends on the activity of the Lecithin Cholesterol Acyl-Transferase (LCAT) which transfer FA from lecithin to cholesterol producing CE. Therefore, the substrate of LCAT is the free cholesterol present in high-density lipoproteins (HDL) [43]. Studies in human and veterinary medicine showed how liver diseases and fatty infiltration into the hepatocytes reduce the secretion and activity of the enzyme. A reduced activity of LCAT therefore results in a decrease of plasma CE [44,45]. In serum of dairy cows, LCAT activity decrease after calving due to excessive hepatic deposition of triglycerides that damage liver function [46]. In the study of Battista et al., dairy cows with liver lipidosis (53.77 ± 43.69 mg/dL) had lower CE concentrations than animals without fatty infiltration (63.06 ± 27.38 mg/dL) [47]. Therefore, ketotic cows reduced blood CE and LCAT concentration compared to healthy cows [36,45]. 

Results of the current study reported lower concentration of FA in ketotic cows (155.86 ± 0.053 mg/dL, NEFA > 0.57 mEq/L) with more lipomobilization and NEB, compared to animals enrolled in NEFA0 group (201.296 ± 0.663 mg/dL, NEFA ≤ 0.57 mEq/L) in accordance with the studies mentioned above.

### 4.3. Effect of Blood NEFA on Lipid Class of PL

Ketotic cows with elevated BHB and NEFA have a plasmatic concentration of PL lower than healthy cows [45]. Yamdagni and Schultz showed that in HK dairy cows, the values of PL decreased by 38% [34]. Indeed, bovine with severe steatosis after calving (4 weeks), resulting from excessive mobilization of fat reserves, have lower concentrations of PL and CE associated with high density lipoproteins (HDL) compared to animals with normal fat infiltration in the liver [36,48]. Van den Top et al., show that Phospholipid Transfer Proteins is positively correlated with LCAT which decrease the concentration immediately postpartum [46]. 

In our study, all significant plasmatic FAs (*p* ≤ 0.05) tended to enhance the concentration with the increases of blood NEFA and BHB (Table 2). The concentration of total FA in ketotic cows (NEFA1, 84.013 ± 28.778 mg/dL) resulted higher than those of the animals with NEFA lower than 0.57 mEq/L (NEFA0, 80.027 ± 31.778 mg/dL) therefore, our data disagreed with what was found in the literature. 

Among the thirty-five FAs identified by TLC-GC, fourteen FAs showed an increasing concentration from healthy (NEFA0) to sick animals (NEFA1) and twenty-one non-significant FAs showed a decreasing concentration, therefore further studies are needed to understand the trend of the phospholipids lipid class.

### 4.4. Effect of Blood NEFA on Lipid Class of TG

In negative energy balance, the influx of NEFAs to the liver exceeds the oxidation capacity of the organ [49]. In addition, metabolic change in the transition period limits the liver’s function of synthesizing and secreting very low-density lipoproteins (VLDL) [49,50,51]. This results in collecting of triglycerides in the hepatocytes defined as steatosis or fatty liver [29]. The liver is a major player in the metabolism of VLDL, the main carrier of TAG [52]. Therefore, the condition of hepatic infarction affects TAG concentrations in plasma [46]. It is known in the available literature that there is a negative correlation between plasma TG and liver TG (r = −0.30) [47]. The drop in TAG concentrations in the blood postpartum may indicate low hepatic synthesis and secretion of VLDL due to the accumulation of TG in the liver [53,54]. Batista et al. showed a negative correlation between plasma triglycerides and serum NEFA (r = −0.39) confirmed by countless studies in which the plasma concentrations of triglycerides decrease in the immediate postpartum, the moment in which the maximum release of NEFA occurs [46,47,52]. Yamdagni and Schultz showed that in HK dairy cows, the values of TG decreased by 53% when compared to concentrations in healthy animals [34].

In the study by Fiore et al., concentration of plasma TG in postpartum cows at 10 DIM is lower than the concentrations of TG at 30 and 50 DIM [55], indeed, the maximum NEB is reached about 14 days after calving [5]. NEFAs have a lipotoxic role in hepatocytes therefore, after the period of maximum NEB, a reduction in the lipolysis process could increase the liver activity for the synthesis of VLDL and it could result in a greater export of TG [51]. In the study of Oikawa et al., the change in the ratio of VLDL-TG to the NEFA, in fasting cows for four days, was assessed. The ratio increases at the beginning of fasting (day 1) and decreases in the period of maximum NEB (day 4). On the tenth day, the ration is four-fold of that on day 0 and this result could explain the elevated transport of liver TG into the blood streaming to enhance steatosis [51]. 

Our study was based on a cross sectional analysis where the samples were conducted at a single point time, however it was not possible to compare the parameters on different lactation days. Decreasing concentrations of triglycerides could have been detected during the first few days of lactation as in the study of Oikawa et al. [51]. Contrary to Batista et al., in our study the negative correlation existing between the plasma concentrations of NEFA and TG is not respected [47]. Dairy cows with high mean values of NEFA and BHB (NEFA1 group) should had a plasma triglyceride concentration lower than animals in a normal state of health (NEFA0 group). The current study, differently from previously cited studies reported that the quantity of total FA increases from healthy subjects (7.105 ± 3.003 mg/dL, NEFA0) to hyperketonemic subjects (56.073 ± 229.531 mg/dL, NEFA1). 

In the study of Gonzalez et al., 2011, concentrations of triglycerides were lower in dairy cows in subclinical ketosis (high BHB) and in animals with high lipid mobilization (high NEFA) [49]. Concentrations of triglycerides lower than 0.12 mmol/L could be used as an indicator of ketosis [56].

In our study, two significant plasmatic FA (*p* ≤ 0.05) of this lipid class tended to increase with the increases of NEFA and BHB concentration (Table 2). Further studies are needed to understand the trend of the different fatty acids in the lipid class of triglycerides as the blood NEFA changes.

### 4.5. Predictive Fatty Acid and Cut-Off Related to Animals in Hyperketonemia

Using the Boruta decisional algorithm, we selected six FAs with predictive value of the state of HK. Four belonged to the lipid class of FFA and two were into the PL fraction. These parameters tended to enhance as lipid mobilization increased, from NEFA0 group to NEFA1 group. Indeed, lipomobilization leads to a change in the acidic profile [9] and a rise in the concentrations of the different FAs in the first lactation phase [39].

The group with predictive values included two monounsaturated fatty acids (MUFAs) specifically Oleic acid (C18:1 ω 9, FFA, AUC = 0.72) and Vaccenic acid (C18:1 ω 7, FFA, AUC = 0.70). Moreover, three saturated fatty acids (SFA) were predictive: Caprylic acid (C8:0, PL, AUC = 0.73) and Lauric acid (C12:0, PL, AUC = 0.78) with a medium hydrocarbon chain and Myristic acid (C14:0, FFA, AUC = 0.77) with long hydrocarbon chain. Finally, only one polyunsaturated fatty acid (PUFA), α-Linolenic acid (C18:3 ω 3, FFA), was predictive. In the class of FFA, concentration of C18:3 ω 3 increased in according to the increase in blood NEFA and BHB. PUFA omega 3 tends to decrease their plasmatic concentration after calving, as they are to a greater extent incorporated into the milk by the mammary gland when the NEB is maximum [39]. The FFA C18:3 ω 3 resulted with a predictive function on the basis of the Boruta algorithm but, the ROC curve analysis revealed a poor diagnostic accuracy. Indeed, AUC of the FFA C18:3 ω 3 was 0.68 and this low value excluded this fatty acid a possible marker of HK.

The FA profiles of cows are affected by the stage of lactation [39]. In early lactation, when the NEB and lipomobilization has maximum expression, MUFA presents the higher plasma concentration compared to dry period or other phase of lactation where the concentration drops [39]. Oleic acid is the predominant fatty acid in adipose tissue and the primary FA released in the blood stream during lipolysis of fat store [26]. The high concentration of C18:1 ω 9 in adipose tissue derives from the activity of delta-9 desaturase (Δ-9 desaturase), which converts C18:0 to C18:1 ω 9 in adipocytes [37]. In our study, C18:1 ω 9 showed increasing concentrations from healthy cows (NEFA0) to NEFA1 group. The threshold value, obtained by ROC analysis and used to discriminate between healthy animals and animals in clinical-subclinical ketosis, is equal to 1.370 mg/dL. In HK the trend of concentration of the FA is variable from one to the other indeed, the C18:1 ω 9 is a biomarker of a state of hyperketonaemia when it exceeds the cut-off. Furthermore, high milk concentration of C18:1 ω 9 could be used as biomarker for diagnosis of high blood concentration of NEFA and BHB in early phase of lactation [38,57]. Concentrations of Oleic acid equal to or greater than 24 g per 100 g of milk indicate the presence of blood NEFA greater than 0.6 mmol/L [57]. C18:0 and C18:1 ω 9 are the main plasma FAs taken from the mammary gland as precursors of milk fat [38]. However, plasma and milk concentrations of Oleic acid cannot be closely correlated as the amount of C18:1 ω 9 milk component derives also from the desaturation of Stearic acid by the Δ-9 desaturase [37,58]. In the study of Lui et al., plasma FA profile has been compared between healthy and hyperketonemic cows (BHB > 1.2 mmol/L) [35]. Dairy cows in ketosis had higher concentrations of MUFA both in prepartum and in postpartum [35]. The study mentioned confirms our results in fact, the predictive MUFA, of the state of hyperketonaemia, C18:1 ω 9 (FFA) and C18:1 ω 7 (FFA), tended to increase concentrations with increasing blood BHB (NEFA1). To diagnose ketosis, the blood concentrations of Vaccine acid in transitional cows, should therefore exceed the threshold value established by ROC analysis equal to 0.082 mg/dL. Lui et al. reported that C18:1 ω 9 had higher concentrations in ketotic cows than in healthy cows and it was used as a biomarker for the diagnosis of the onset of ketosis [35].

The SFA profile of cows is affected by the stage of lactation and in particular in the lactating period there is a decrease in concentration value compared to the dry period [39]. Furthermore, in the transition period, ketosis cows have lower SFA concentrations than cows with normal blood BHB [35]. Contrary to the mentioned study, our results reported that C8:0 (PL, AUC = 0.73), C12:0 (PL, AUC = 0.78) and C14:0 (FFA, AUC = 0.77), showed an increase in contractions from healthy cows (NEFA0, 0.019 ± 0.017 mg/dL, 0.479 ± 0.215 mg/dL and 0.204 ± 0.05 mg/dL) to sick cows (NEFA1, 0.095 ± 0.027 mg/dL, 1649 ± 0.340 mg/dL and 0.517 ± 0.071 mg/dL). The divergence of our results from the data reported in the literature, could be explained by the different type of absorption that exists between medium hydrocarbon chain fatty acids (MCFAs) and LCFAs. MCFAs do not require the action of bile salts nor the absorption mediated by micelles and chylomicrons contrary to what occurs for LCFA absorption [59]. The increase in SFA, consisting of medium hydrocarbon chain, could derive from the absorption without bile salts which decreases in conditions of liver lipidosis [15]. 

The high concentration of NEFA and the variation of mean value of the different fatty acids increase the risk of inflammatory diseases such as mastitis and metritis because the composition of fatty acids in the membranes of the main cells of the immune system are altered, thus decreasing the functionality of immune cells [10,60,61]. Therefore, identification of fatty acids in the different lipid classes is important both as an early diagnostic tool of metabolic disease and as a diagnostic method of immunosuppression in dairy cow.

## 5. Conclusions

In conclusion, different plasma FA profiles between healthy cows and animals with elevated NEFA could be used for an early detection of HK in transition animals. The greatest predictive function was associated with the FFA and PL fractions. C18:1 ω 9 was the main monounsaturated fatty acid with high predictive value. A plasma concentration higher than 1.370 mg/dL has a good tool in diagnosing of HK. 

In the PL class, C12:0 demonstrated a good diagnostic function. A concentration of C12:0 higher than 0.567 mg/dL permits to discriminate between healthy and hyperketonemic cows.

In the future, it would be interesting to investigate the changes in the plasmatic concentrations of FA during the whole transition period, from the last week of gestation to seventh week of lactation. Further studies are needed to evaluate the existing correlation with metabolic disorder, infectious pathologies, the phenotypic characteristics and reproductive performance. Plasma fatty acids could be used as a biomarker for countless diseases and phenotypic traits using TLC-GC as tool diagnostic for the evaluation of dairy cow in the transition period.

## Figures and Tables

**Table 1:** Mean value (±SD) of non-esterified fatty acid (NEFA), β-hydroxybutyrate (BHB), days in milk (DIM), body condition score (BCS), parity and daily milk yield.

Parameters	NEFA0	NEFA1	Correlation (*p*-Value)
NEFA (mEq/L)	0.24 ± 0.12	0.87 ± 0.23	0.001
BHB (mmol/L)	0.65 ± 0.28	1.21 ± 0.28	0.001
DIM	28.25 ± 13.75	26.68 ± 15.49	NS
BCS	2.78 ± 0.21	2.89 ± 0.15	NS
N parity	2.68 ± 1.80	2.57 ± 1.40	NS
Milk (Kg/day)	29.55 ± 7.87	30.24 ± 8.44	NS

NEFA: non esterified fatty acid; BHB: β-hydroxybutyrate; DIM: day in milk; BCS: body condition score; NS: not significant.

**Table 2:** Mean value of plasma fatty acids (±SD) relating to the lipid class of free fatty acids (FFAs), cholesterol esters (CE), phospholipids (PL) and triglycerides (TG) in relation to two classes of NEFA (NEFA0–NEFA1).

Fatty Acids	FFA		CE		PL		TG	
	NEFA 0	NEFA 1	NEFA 0	NEFA 1	NEFA 0	NEFA 1	NEFA 0	NEFA 1
C8	0.375 ± 0.093	0.709 ± 0.132 *	1.431 ± 0.095	1.240 ± 0.13	0.019 ± 0.017	0.095 ± 0.027 *	0.046 ± 0.015	0.079 ± 0.022
C10	0.143 ± 0.049	0.248 ± 0.069	0.803 ± 0.059	0.691 ± 0.081	0.093 ± 0.017	0.193 ± 0.027 **	0.075 ± 0.026	0.157 ± 0.039 *
C12	0.643 ± 0.177	1.168 ± 0.251	2.934 ± 0.361	2.023 ± 0.496	0.479 ± 0.215	1.649 ± 0.340 **	0.737 ± 0.099	0.894 ± 0.144
C14	0.204 ± 0.05	0.517 ± 0.07 ***	1.229 ± 0.183	0.974 ± 0.251	0.298 ± 0.102	0.798 ± 0.161 *	0.275 ± 0.052	0.371 ± 0.076
C14:1 ω 5	0.051 ± 0.009	0.096 ± 0.012 **	0.745 ± 0.115	0.551 ± 0.158	0.191 ± 0.051	0.091 ± 0.080	0.059 ± 0.008	0.070 ± 0.012
C16	1.365 ± 0.257	2.908 ± 0.364 **	10.183 ± 0.966	9.476 ± 1.325	13.909 ± 1.019	14.692 ±1.611	1.508 ± 0.161	1.709 ± 0.234
C16:1ω7	0.056 ± 0.026	0.172 ± 0.037 *	1.714 ± 0.228	2.343 ± 0.313	0.356 ± 0.038	0.402 ± 0.060	0.027 ± 0.010	0.054 ± 0.015
C18	1.176 ± 0.201	2.315 ± 0.285 **	1.628 ± 0.151	1.380 ± 0.208	16.247 ± 1.293	15.969 ± 2.044	1.532 ± 0.160	1.666 ± 0.233
C18:1 ω 9	0.456 ± 0.448	3.241 ± 0.634 ***	5.489 ± 0.724	7.085 ± 0.994	9.427 ± 0.883	11.484 ± 1.397	0.290 ± 0.056	0.366 ± 0.081
C18:1 ω 7	0.045 ± 0.025	0.204 ± 0.036 ***	0.671 ± 0.063	0.610 ± 0.087	1.306 ± 0.134	1.221 ± 0.211	0.045 ± 0.008	0.050 ± 0.011
C18:2 ω 6	0.302 ± 0.049	0.475 ± 0.069 *	146.923 ± 16.167	103.919 ± 22.181	21.347 ± 1.636	19.148 ± 2.587	0.310 ± 0.045	0.360 ± 0.065
C18:3 ω 6	0.013 ± 0.003	0.018 ± 0.004	2.846 ± 0.501	1.61 ± 0.687	0.300 ± 0.041	0.282 ± 0.064	0.076 ± 0.019	0.089 ± 0.028
C18:3 ω 3	0.011 ± 0.004	0.031 ± 0.005 **	10.380 ± 1.07	8.784 ± 1.468	1.328 ± 0.137	1.226 ± 0.216	0.018 ± 0.004	0.009 ± 0.006
C18:4 ω 3	0.213 ± 0.031	0.221 ± 0.043	1.538 ± 0.276	1.201 ± 0.379	0.377 ± 0.101	0.258 ± 0.160	0.241 ± 0.039	0.302 ± 0.057
C20	0.017 ± 0.006	0.032 ± 0.009	0.098 ± 0.016	0.078 ± 0.022	0.103 ± 0.010	0.096 ± 0.016	0.035 ± 0.015	0.080 ± 0.021 *
C20:1 ω 9	0.006 ± 0.003	0.014 ± 0.004	0.028 ± 0.015	0.041 ± 0.02	0.082 ± 0.010	0.086 ± 0.016	0.005 ± 0.002	0.006 ± 0.003
C20:1 ω 7	0.011 ± 0.004	0.024 ± 0.00 5*	0.080 ± 0.020	0.075 ± 0.027	0.080 ± 0.011	0.111 ± 0.018	0.016 ± 0.004	0.021 ± 0.005
C20:2 ω 6	0.056 ± 0.025	0.095 ± 0.035	0.573 ± 0.866	2.866 ± 1.188	0.235 ± 0.036	0.209 ± 0.057	0.124 ± 0.042	0.214 ± 0.061
C20:3 ω 9	0.029 ± 0.011	0.015 ± 0.016	0.114 ± 0.342	1.144 ± 0.469 *	0.070 ± 0.012	0.042 ± 0.020	0.099 ± 0.034	0.147 ± 0.050
C20:3 ω 6	0.008 ± 0.002	0.015 ± 0.002 *	0.244 ± 0.058	0.292 ± 0.08	2.369 ± 0.261	2.247 ± 0.413	0.017 ± 0.003	0.015 ± 0.004
C20:4 ω 6	0.012 ± 0.005	0.012 ± 0.008	1.256 ± 0.303	1.595 ± 0.416	2.853 ± 0.262	3.486 ± 0.415	0.007 ± 0.002	0.012 ± 0.003
C20:3 ω 3	0.008 ± 0.002	0.009 ± 0.003	0.104 ± 0.033	0.032 ± 0.045	0.035 ± 0.004	0.022 ± 0.007	0.024 ± 0.007	0.021 ± 0.009
C20:4 ω 3	0.148 ± 0.024	0.173 ± 0.034	0.567 ± 0.103	0.484 ± 0.141	0.443 ± 0.077	0.223 ± 0.122	0.188 ± 0.033	0.262 ± 0.048
C20:5 ω 3	0.016 ± 0.006	0.026 ± 0.008	0.933 ± 0.133	0.931 ± 0.183	0.544 ± 0.058	0.653 ± 0.091	0.007 ± 0.002	0.008 ± 0.003
C22	0.011 ± 0.003	0.012 ± 0.004	0.155 ± 0.034	0.113 ± 0.047	0.723 ± 0.054	0.685 ± 0.085	0.019 ± 0.005	0.022 ± 0.008
C22:1 ω 9	0.006 ± 0.002	0.005 ± 0.002	0.042 ± 0.006	0.043 ± 0.008	0.008 ± 0.002	0.008 ± 0.004	0.017 ± 0.003	0.018 ± 0.004
C22:2 ω 6	0.009 ± 0.002	0.009 ± 0.003	0.059 ± 0.010	0.049 ± 0.014	0.114 ± 0.034	0.057 ± 0.054	0.008 ± 0.005	0.007 ± 0.007
C22:4 ω 6	0.055 ± 0.007	0.039 ± 0.010	0.216 ± 0.039	0.203 ± 0.053	0.551 ± 0.061	0.377 ± 0.096	0.052 ± 0.010	0.068 ± 0.014
C22:5 ω 3	0.012 ± 0.004	0.007 ± 0.005	1.600 ± 1.280	0.078 ± 1.756	1.872 ± 0.493	1.194 ± 0.780	0.006 ± 0.002	0.008 ± 0.003
C22:6 ω 3	0.020 ± 0.005	0.018 ± 0.007	0.094 ± 0.015	0.061 ± 0.02	0.273 ± 0.054	0.130 ± 0.086	0.02 ± 0.009	0.008 ± 0.013
C23	0.007 ± 0.001	0.006 ± 0.002	0.017 ± 0.005	0.017 ± 0.007	1.327 ± 0.119	1.153 ± 0.188	0.005 ± 0.002	0.008 ± 0.002
C24	0.018 ± 0.003	0.012 ± 0.005	0.171 ± 0.028	0.113 ± 0.038	0.948 ± 1.124	4.011 ± 1.778	0.008 ± 0.003	0.009 ± 0.004
C24:1 ω 9	0.034 ± 0.008	0.036 ± 0.012	0.106 ± 0.017	0.060 ± 0.023	0.439 ± 0.065	0.374 ± 0.103	0.014 ± 0.005	0.010 ± 0.008
C16 DMA	0.354 ± 0.026	0.387 ± 0.037	5.860 ± 0.479	5.252 ± 0.658	0.386 ± 0.051	0.311 ± 0.081	0.883 ± 0.759	2.930 ± 1.103
mg FA/dL	5.960 ± 2.852	13.369± 10.591	201.296± 41.663	155.86± 32.053	80.027 ± 31.778	84.013 ± 28.778	7.105± 3.003	56.073± 229.531
mg/dL	5.960 ± 2.852	13.369 ± 10.591	471.808 ± 248.588	364.572 ± 184.57	110.367 ± 44.440	115.948 ± 51.196	7.484 ± 3.606	60.21 ± 209.531

*: *p*-Value ≤ 0.05; **: *p*-Value ≤ 0.01; ***: *p*-Value ≤ 0.001.

**Table 3:** Mean, median, minimum value, maximum value and normal value of importance of the 6 predictive fatty acids (FAs).

Fatty Acids	MeanImp	MedianImp	MinImp	MaxImp	NormHits	Decision
C18:1 ω 9 (FFA)	10.823	11.444	3.923	13.783	1.000	Confirmed
C18:1 ω 7 (FFA)	6.800	7.029	3.208	9.252	0.959	Confirmed
C8:0 (PL)	8.043	8.664	1.925	11.119	0.939	Confirmed
C18:3 ω 3 (FFA)	4.786	4.834	1.936	6.759	0.848	Confirmed
C12:0 (PL)	5.061	5.230	−0.639	8.609	0.788	Confirmed
C14:0 (FFA)	3.734	3.809	0.948	6.064	0.666	Confirmed

MeanImp: mean of the importance value; MedianImp: median of the importance value; MinImp: minimum of the importance value; MaxImp: maximum of the importance value; NormHits: value of normalized importance.

**Table 4:** Results of the ROC analysis of the predictive fatty acids (FAs) on the basis of NEFA values (greater than 0.56 mEq/L).

Fatty Acids	Cut-Off (mg/dL)	AUC	Sensitivity (Se)	95% IC for Se	Specificity (Sp)	95% IC for Sp	*p*-Values
C12:0 (PL)	>0.567	0.78	75.00	42.8–94.5	80	61.4–92.3	<0.0001
C14:0 (FFA)	>0.167	0.77	88.89	65.3–98.6	52.78	35.5–69.6	<0.0001
C8:0 (PL)	>0.035	0.73	58.33	27.7–84.8	93.33	77.9–99.2	<0.0001
C18:1ω9 (FFA)	>1.370	0.72	61.11	35.7–82.7	94.44	81.3–99.3	<0.0001
C18:1ω7 (FFA)	>0.082	0.70	55.56	30.8–78.5	86.11	70.5–95.3	<0.0001
C18:3ω3 (FFA)	>0.032	0.68	44.44	21.5–69.2	94.44	81.3–99.3	<0.0001

AUC: Area Under the Curve.
